# Microwave power penetration enhancement inside an inhomogeneous human head

**DOI:** 10.1038/s41598-021-01293-4

**Published:** 2021-11-08

**Authors:** Md. Rokunuzzaman, Asif Ahmed, Thomas Baum, Wayne S. T. Rowe

**Affiliations:** School of Engineering, RMIT University, Melbourne, VIC 3001 Australia

**Keywords:** Electrical and electronic engineering, Cancer imaging

## Abstract

The penetration of microwave power inside a human head model is improved by employing a dielectric loaded rectangular waveguide as the transmission source. A multi-layer reflection model is investigated to evaluate the combined material characteristics of different lossy human head tissues at 2.45 GHz. A waveguide loaded with a calculated permittivity of 3.62 is shown to maximise the microwave power penetration at the desired frequency. A Quartz (*SiO*_2_) loaded rectangular waveguide fed by a microstrip antenna is designed to validate the power penetration improvement inside an inhomogeneous human head phantom. A measured 1.33 dB power penetration increment is observed for the dielectric loaded waveguide over a standard rectangular waveguide at 50 mm inside the head, with an 81.9% reduction in the size of the transmission source.

## Introduction

Over the past decade, microwave human body diagnostic system development has gained a great deal of interest among researchers due to its potential in biomedical sector, specifically in microwave biomedical imaging (MBI) applications^1–4^. Depending on the dielectric properties of human tissues, MBI system sensitivity may vary^5^. To determine unknown malignant tissues (e.g. tumour, haemorrhage) inside the human body, the microwave material characteristics of human tissues must be known^6^.

An investigation into the optimal range of frequencies for power penetration inside different dispersive tissues of the human body has previously been conducted^7^. For a cluster of human head tissues, the ideal operational frequency is reported to range between 0.5 to 2.5 GHz^1,6,8^. A key challenge in MBI is to achieve sufficient power penetration inside dispersive human tissue at these frequencies^9^, particularly at the higher end of the range. Unlike other imaging systems, human head imaging utilizing microwave technology must consider the rate of energy absorbed by the human tissue when exposed to an electromagnetic field, known as specific absorption rate (SAR), which limits the power that can be radiated from a transmission source. Moving the transmitter away from the head can relax the limitation imposed by SAR, but consequently more than half of the total energy radiated can reflect back at the air-skin interface^4^, diminishing power penetration inside the human brain. Hence, many radiating elements designed for MBI lack power efficiency^1,2,6^. The physical distance between the radiating element and the skin introduce further loss to the overall system. The resulting reflections from the malignant tissue (which can be a significant distance inside the human brain) become extremely weak and clouded by other unwanted reflections, making retrieval of information from the signal problematic. There are two kinds of antennas reported in the literature for MBI, namely off-body and on-body antennas. Antennas which operate off-body suffer from low front to back (FBR) ratio^2,10–14^ largely due to the reflection from the high dielectric permittivity bio-tissue skin.

To mitigate this problem, on-body antennas can be designed. Only a few on-body antennas are reported in the literature for MBI applications^15^, however these examples consider a homogeneous human head phantom for simulation and measurement. An on-body matched antenna can be designed on a high permittivity substrate materials, which leads to smaller antenna dimensions^4^. Although the antenna reflection performance is better when in contact with skin, it is hard to determine the power penetration inside the dispersive tissue due to lack of evidence. In^16^, an antenna designed for free space operation is applied on top of human tissue with an impedance matching layer in between. It is shown that by using a matching layer with the same permittivity of the bulk human tissue can provide higher power penetration. The matching layer is assumed to be lossless and the antenna is operating in air next to the matching layer. In reality, it is challenging to find material with such characteristics to use as matching layer. Moreover, the human tissue has an inhomogeneous structure which is not taken into consideration while introducing the matching layer.

The most disadvantageous feature of microwave diagnostics of the head compared to other parts of the human body is the high water content of brain tissue^17^. Since the brain is more than 75% water, most of the microwave power penetrating through the outer boundary of human head suffers from high loss. Hence, it is crucial to achieve efficient transfer of energy through the boundary of the head to achieve a sufficient level of power penetration inside the brain.

In this paper, microwave power penetration inside a human head phantom is improved by matching the radiation source to the impedance faced by a wave propagating through inhomogeneous layered tissue materials. A multi-layer reflection model is considered to estimate the combined material characteristic of the different tissue layers (i.e. skin, bone, brain) at 2.45 GHz. The impedance of a wave in a rectangular waveguide is modified utilizing a low loss material, forming a dielectric loaded waveguide to match to the inhomogeneous layered head phantom. The power penetration at different depths inside human brain is evaluated and compared to the unmatched case. To validate the results, a Quartz (*SiO*_2_) loaded waveguide is constructed and fed via a microstrip antenna. Human tissue mimicking inhomogeneous layers are fabricated and utilized as a human head phantom to obtain the measurement results.

## Multilayer wave impedance formulation

**Figure 1:**
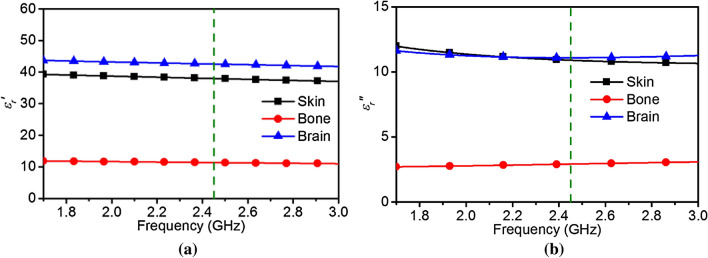
(**a**) Real and (**b**) imaginary permittivity of human skin, bone and brain (derived from^18^). The dashed lines indicate the selected frequency.

The human head consists of a number of lossy dielectric media, but can be approximated with a combination of inhomogeneous layers (i.e. skin, bone, brain) to conceive a model with greater rigor than a bulk approximated phantom as used in^16^. Such a layered structure needs to be formulated to understand the wave propagation throughout the layers before utilizing them for a MBI system. Microwave propagation in a media can be defined by the wave impedance characteristics. For lossy media, the wave impedance can be expressed as a complex impedance where the imaginary part of the impedance represents the loss in the media. To attain the wave impedance, the dielectric properties (i.e. permittivity, permeability) at the desired frequency must be known. The complex permittivity parameters for different biological tissues can be found in^18^. The real and imaginary parts of the permittivity for the considered bio-tissues are depicted in Fig. 1. For this work, a frequency *f*_1_ = 2.45 GHz is chosen as it is close to the upper bound of the optimal range for human head tissues. A simplified inhomogeneous head model consisting of three layers (i.e. skin, bone, brain) is considered.

The wavelength *λ*_*x*_ for frequency *f*_1_ in different biological tissue can be calculated using:

1 $$\lambda_{x} = \frac{{c_{0} }}{{f_{1} \sqrt {\varepsilon_{x} } }}$$

To characterize the wave propagation inside the layered biological tissues at frequency *f*_1_, the reflections occurring at each interface can be combined to achieve overall reflection coefficient utilizing:

2 $$\Gamma_{overall} \cong \Gamma_{0} + \sum \Gamma_{x} e^{ - j2x\theta }$$

where, $\theta = \frac{2\pi }{{\lambda_{x} }}d_{x}$ for each layer with tissue thickness *d*_*x*_, and $\Gamma_{0}$ is the reflection coefficient at the boundary of air and skin. The specific reflection coefficient at the interface between different materials can be found using the normalized Fresnel equation for reflection:

3 $$\Gamma_{x} = \frac{{\eta_{x + 1} - \eta_{x} }}{{\eta_{x + 1} + \eta_{x} }}$$

where,

4 $$\eta_{x} = \frac{{\eta_{0} }}{{\sqrt {\varepsilon_{x} } }}$$

$\eta_{0}$ is the wave impedance of free space, $\eta_{x}$ and $\eta_{x + 1}$ are the impedances of the incident and transmitted wave materials respectively. For the considered tissue materials, the wave impedances can be found in Table 1.

**Table 1:** Human tissue characteristics at 2.45 GHz^18^.

Material	Complex permittivity (ε′ + jε″)	Considered thickness d_x_ (mm)	Wave impedance, η_x_ (Ω)
Skin	38.01 + j10.88	1	59.37 − j8.34
Bone	11.36 + j2.916	10	109.18 − j13.78
Brain	42.55 + j11.08	60	56.39 − j7.22

Once the overall reflection coefficient is found using (2), the wave impedance of the overall layered biological structure $\eta_{overall}$ can be found using the general form of the reflection coefficient equation:

5 $$\eta_{overall} = - \eta_{0} \left( {\frac{{1 + \frac{1}{{\Gamma_{overall} }}}}{{1 - \frac{1}{{\Gamma_{overall} }}}}} \right)$$

As a rectangular waveguide is to be used as the radiation source, the wave impedance of the dominant TE_10_ mode can be determined using:

6 $$\eta_{TE} = \frac{{\left| {\eta_{overall} } \right|}}{{\sqrt {1 - \left( {\frac{{f_{c} }}{{f_{1} }}} \right)^{2} } }}$$

where *f*_*c*_ is the cut-off frequency of the waveguide.

Owing to the irremovable conductivity of the biological tissues, the conductive material loss of a propagating wave inside the human head cannot be minimized. However, by matching $\eta_{TE}$ with $\eta_{overall}$ at the outer boundary of the layered biological structure, maximum power penetration inside human head is possible. This matching can be achieved by utilizing a dielectric loaded rectangular waveguide filled with a material of permittivity, *ε*_*wg*_. Utilizing the wave impedance of the TE wave in (6), the *ε*_*wg*_ required can be found from:

7 $$\varepsilon_{wg} = \left( {\frac{{\left| {\eta_{overall} } \right|}}{{\eta_{TE} }}} \right)^{2}$$

By utilizing the equations, the dielectric loaded rectangular waveguide permittivity *ε*_*wg*_ is found to be 3.6 − j5.8 at 2.45 GHz. The imaginary part of the permittivity contributes to the loss of the material, which if included in the waveguide dielectric material will introduce more loss to the overall system. Thus utilizing just the real part of the permittivity *ε*_*wg*_ becomes 3.6 at 2.45 GHz.

## Simulation setup

**Figure 2:**
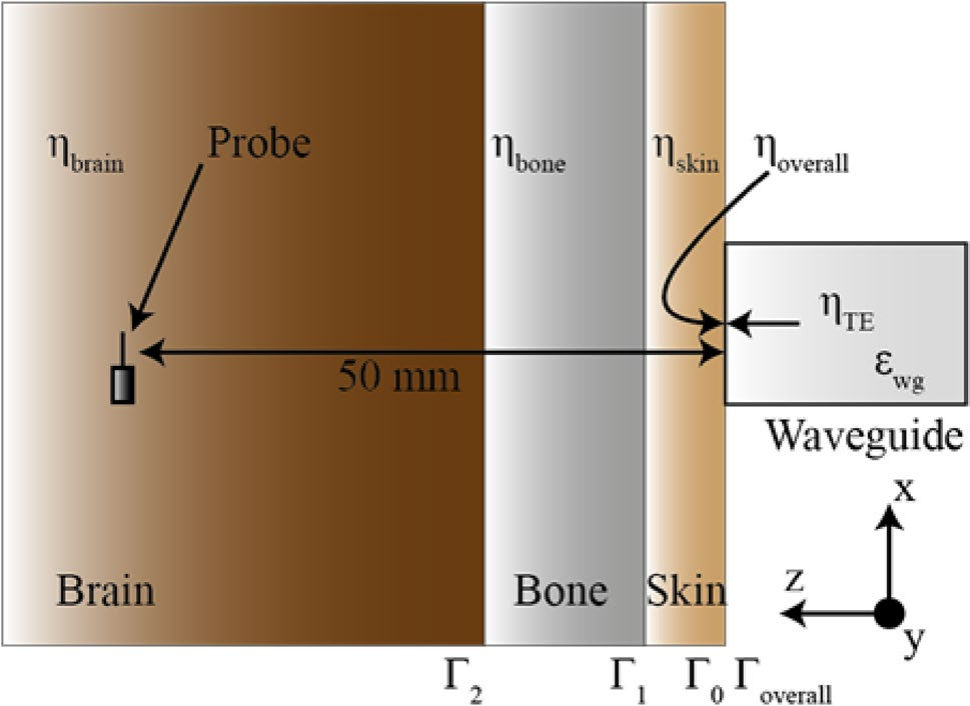
Experimental setup—rectangular waveguide aperture against an inhomogeneous human head phantom.

To evaluate the validity of the calculated result, a Computer Simulation Technology (CST) Microwave Studio^19^ simulation setup as depicted in Fig. 2 is adopted. The complex permittivities and thicknesses of the human tissue materials are set to those stated in Table 1. The broad wall dimension of the dielectric filled rectangular waveguide, *a*, is calculated via:

8 $$f_{{cTE_{10} }} = \frac{c}{{2a\sqrt {\mu_{m} \varepsilon_{m} } }}$$

where *f*_*c*_ is the selected cut-off frequency, *μ*_*wg*_ and *ε*_*wg*_ are the permeability and permittivity of the dielectric filling material, and *c* is the speed of light in free space. Once the *a* is found, the short wall dimension *b* is found as:

9 $$b \le \frac{a}{2}$$

A cut-off frequency of 1.7 GHz was selected to mimic that of an air filled WR430 waveguide, and the dimensions are found to be *a* = 47 mm and *b* = 23 mm*.*

**Figure 3:**
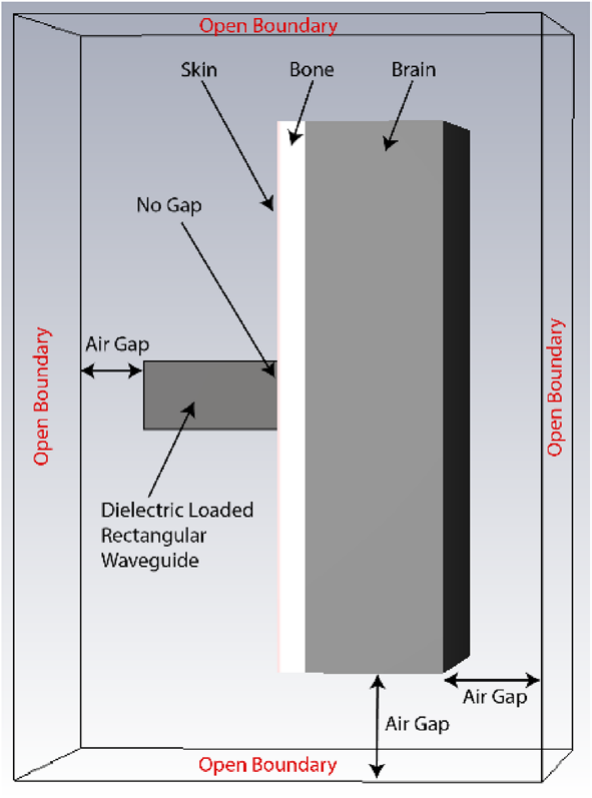
Simulation setup of the open-ended waveguide-phantom interface.

In the CST 3D schematic shown in Fig. 3, the open end of the quartz loaded rectangular waveguide is attached directly to the surface of the skin layer. There is no air gap, or any other material considered at the interface to the phantom. An open boundary with added space condition from all sides of the phantom and waveguide is considered for simulation.

**Figure 4:**
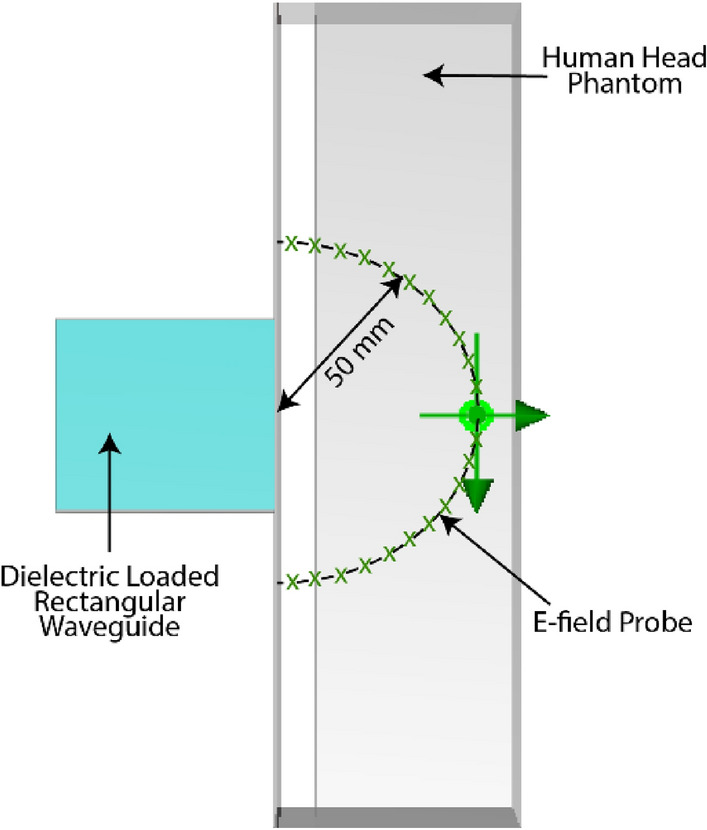
Top view of the E-field probe setup at a 50 mm radius inside the human head model from the center of the waveguide-skin interface.

Multiple E-field probes within the CST simulator are placed inside the human phantom at 50 mm radius from the epicenter of the waveguide-phantom interface to achieve a measure of the E-field intensity at 50 mm inside the head model from the waveguide-skin interface (Fig. 4).

## Results and discussion

**Figure 5:**
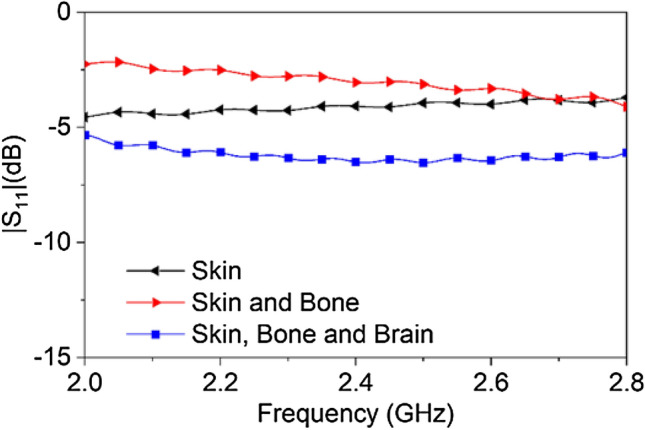
Reflection coefficient at the antenna port with the addition of different layers of human body phantom materials at the opening of the dielectric loaded rectangular waveguide.

Figure 5 shows the reflection coefficient of three different setups; i.e. (1) a phantom consisting of only the skin layer, (2) skin and bone, and (3) skin, bone and brain attached to the open end of the dielectric loaded waveguide respectively. The reflection coefficient shows the lowest response when all tissue layers (skin → bone → brain) are attached together at the interface as the dielectric loaded waveguide is designed to interface to this particular configuration of the human head phantom.

**Figure 6:**
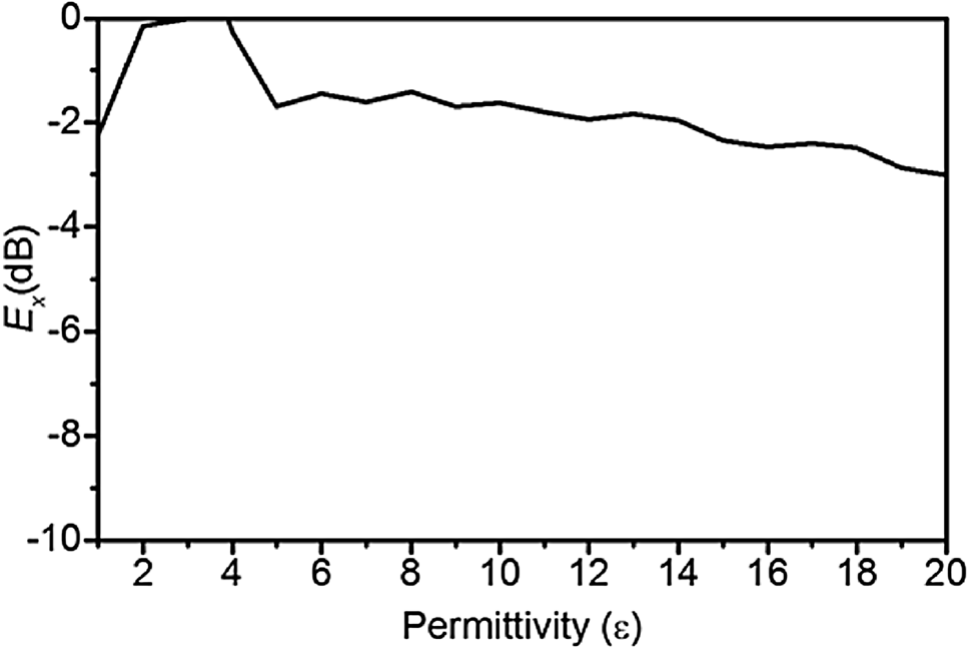
Normalized *E*_*x*_-field intensity at 50 mm inside human head versus dielectric filled waveguide permittivity variation at 2.45 GHz.

The simulated normalized *x*-directed electric-field (*E-field*, *E*_*x*_) penetration at 2.45 GHz is shown in Fig. 6 at 50 mm inside the head model from the surface of the skin for different permittivity dielectric loaded rectangular waveguides. For each change of permittivity, the waveguide dimensions were changed accordingly to maintain the same cut-off frequency. The maximum field penetration occurs when the rectangular waveguide dielectric permittivity, *ε*_*wg*_, is within the range of 3 to 4.

**Figure 7:**
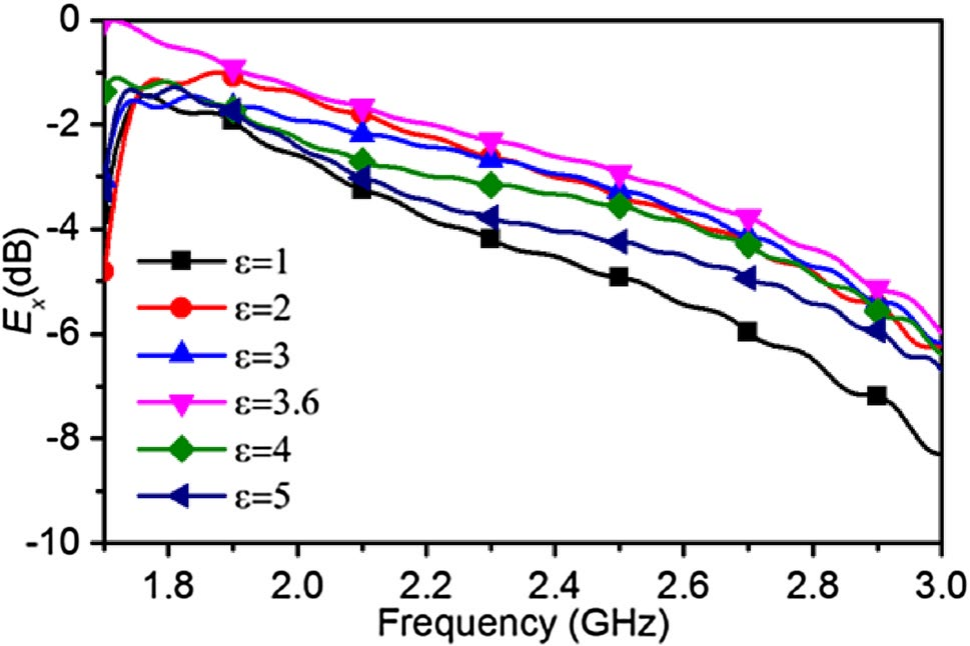
Normalized *E*_*x*_-field intensity at 50 mm inside human head versus dielectric filled waveguide permittivity variation from 1.7 to 3 GHz.

A frequency sweep from 1.7 to 3 GHz in Fig. 7 shows the normalized *E*_*x*_ penetration performance for different *ε*_*wg*_ from 1 to 5. The maximum E-field penetration occurs at *ε*_*wg*_ = 3.6 over the entire range, but is particularly pronounced at the lower frequencies. Although the dielectric characteristics of human head may change slightly with frequency, it can be observed that over the small range of frequencies of interest the E-field penetration is maximised at the calculated value of 3.6. The penetration is primarily minimum when the permittivity of air (*ε*_*air*_ = 1) is used inside the waveguide.

**Figure 8:**
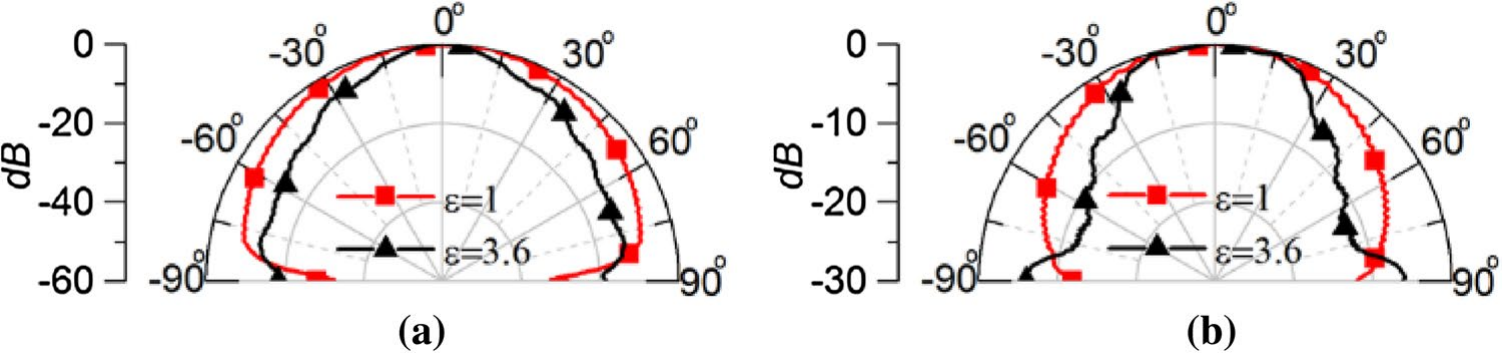
Simulated normalized (**a**) *E-plane* (*xz*-plane) and (**b**) *H-plane* (*yz*-plane) near-field pattern at 50 mm distance inside human brain.

Figure 8 portrays the simulated normalized *E-plane* (containing the electric field vector) and *H-plane* (containing the magnetic field vector) near field patterns at 50 mm distance inside the human head for the waveguide dielectric filling of 1 and 3.62 respectively. The resulting radiation beam inside the brain is highly directional when permittivity of 3.62 is used compared to the air-filled waveguide. Hence improved directivity inside the human brain is achieved by using the optimized dielectric filled waveguide.

**Figure 9:**
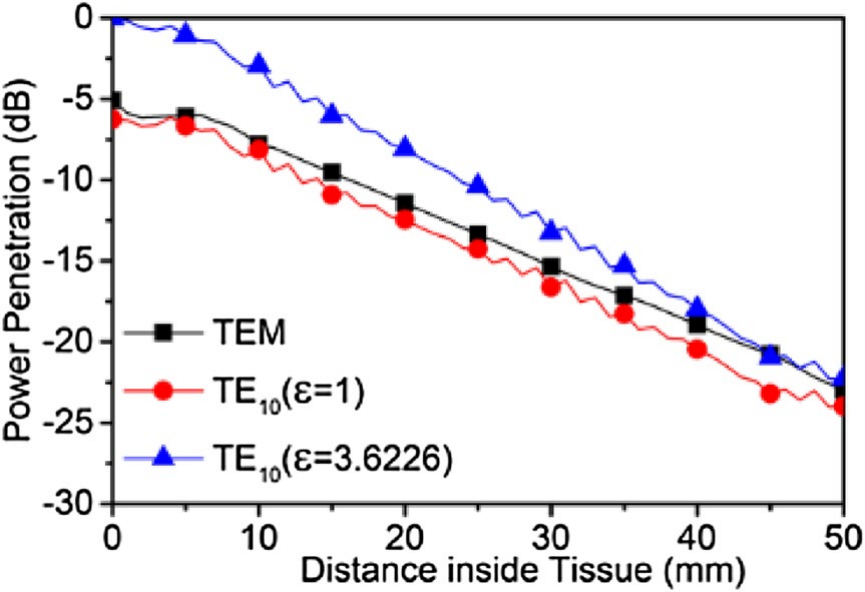
Power penetration of TEM and TE_10_ radiated waves up to 50 mm inside human head at 2.45 GHz.

The power penetration $\vec{P}$ in the propagation direction at a specific location can be calculated using the cross product of the $\overrightarrow {E }$ and $\vec{H}$-field at a specific frequency. To observe the different transverse mode propagation characteristics inside the human head, Fig. 9 compares the power penetration for a TEM plane wave with TE_10_ waves radiating from both an air-filled waveguide and an *ε*_*wg*_ = 3.6 dielectric filled waveguide. The highest penetration up to a 50 mm distance inside human head occurs when radiated from the dielectric loaded waveguide. The TE_10_ wave emanating from the air-filled waveguide exhibits 3–7 dB less penetration and is also lower than TEM propagating wave from air into the human head. The loss due to the high-water content of the brain and other tissues can be observed from the penetration power level difference of around 20 dB between 0 and 50 mm inside the head.

**Figure 10:**
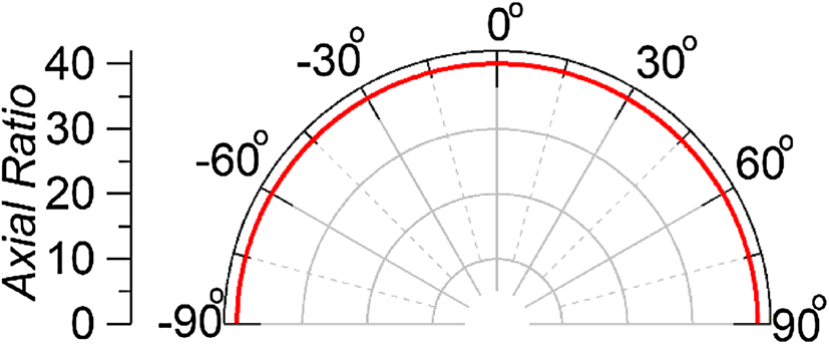
Axial ratio for the open-ended waveguide at 2.45 GHz.

Apart from the dominant mode, higher order modes appear evanescent and hence do not extend any substantial distance from the edge of the open-ended waveguide and hence do not have significant effect on the field measured inside the human brain. Figure 10 portrays the simulated axial ratio of the dielectric loaded rectangular waveguide. An axial ratio of 40 dB is achieved indicating a negligible amount of cross polarized fields at 2.45 GHz.

**Figure 11:**
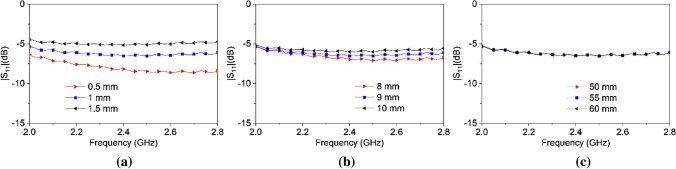
Reflection coefficient of the dielectric loaded rectangular waveguide for different thicknesses of (**a**) Skin, (**b**) Bone, and (**c**) Brain.

As human head tissue thicknesses vary between people, it is imperative to verify the behavior of the waveguide considering this difference. Figure 11 shows the reflection coefficient of the dielectric loaded rectangular waveguide for different thicknesses of each human head phantom material as compared to the baseline value in Table 1. For a 0.5 mm thickness difference in the skin layer, the reflection coefficient shows a determinate amount of variation. The reflection coefficient becomes as low as − 8.44 dB at 2.45 GHz for 0.5 mm skin thickness. With the increment in thickness of the skin, the reflection coefficient become comparatively higher at 2.45 GHz amounting to − 6.4 dB and − 5.02 dB for 1 mm and 1.5 mm skin thicknesses respectively. As the high dielectric constant skin layer is the only layer that is in direct contact with the open end of the waveguide, it is intuitive that it would be sensitive to normal human variation. Figure 11b shows the reflection coefficient with changes in bone thickness of human head phantom. Reflection coefficient variation due to a bone thickness is not as significant as compared to skin thickness. The change in thickness of the brain layer is negligible for increments of 5 mm as shown in Fig. 11c.

To validate the performance described in the previous section, an inhomogeneous human head phantom is fabricated. The composition of tissue mimicking composites used in the fabrication process are shown in Table 2. For the oil concentration, a mixture of 50% Kerosene oil and 50% Safflower oil is used. The procedure presented in^20^ is utilized to make tissue-mimicking layers of the desired permittivity and loss tangent (*tanδ*). To achieve the same permittivity as the skin, bone and brain, an oil percentage of 32%, 70% and 28% were used according to^20^. The characteristics of the phantom tissues fabricated for this experiment maintain their properties for at least two months.

**Table 2:** Tissue mimicking material composition.

Human tissue	Skin	Brain	Bone
Oil (%)	28.4425	23.955	65.2925
Water (%)	57.02125	60.92	25.855
2-Propanol (%)	2.39625	2.56	1.088125
p-Toluic acid (%)	0.059875	0.064	0.0278125
Gelatin (%)	10.20125	10.89875	4.630625
Formaldehyde Sol. (%)	0.24125	0.2575	0.1085
Surfactant (%)	1.63875	1.33375	2.993125

**Figure 12:**
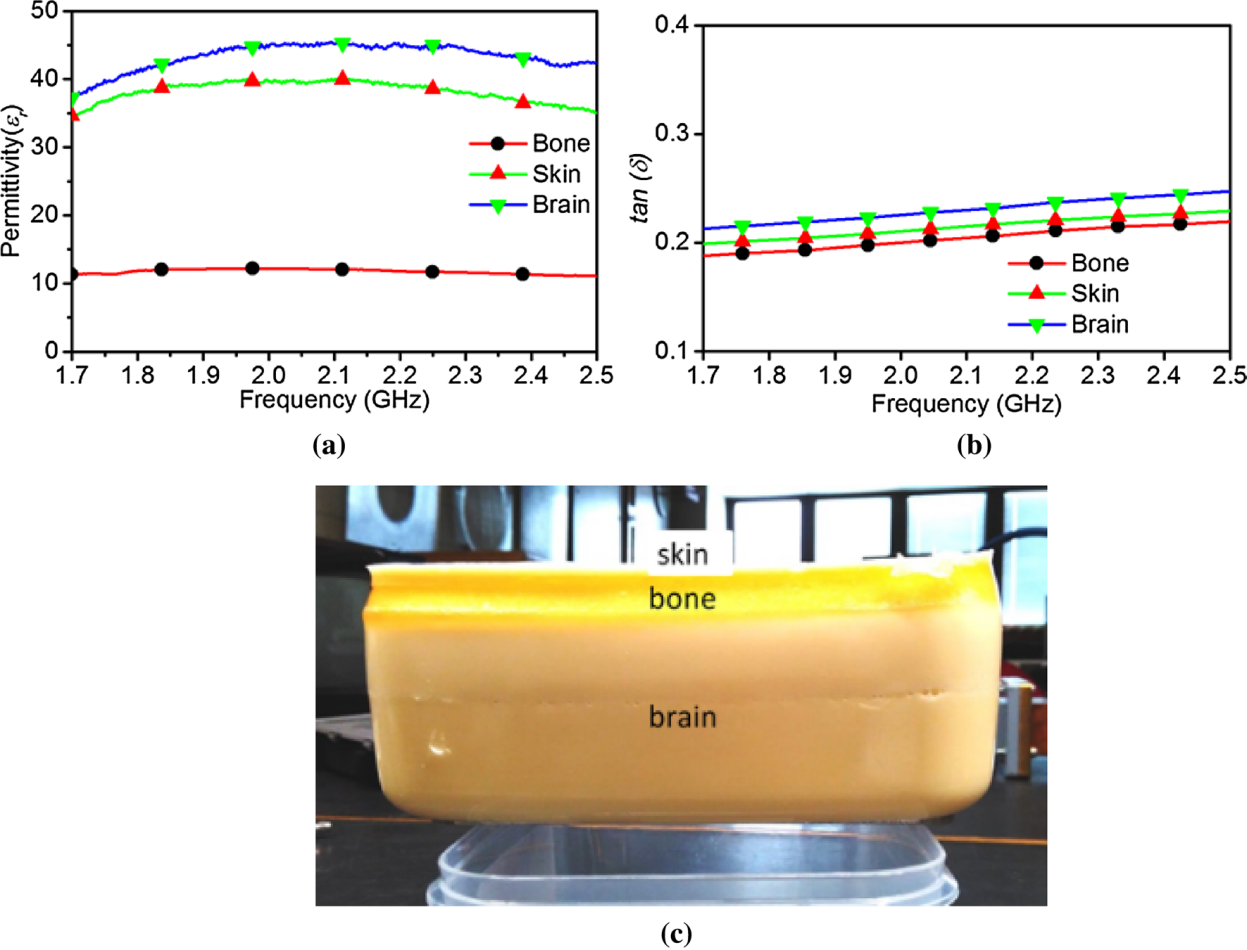
Inhomogeneous human head phantom (**a**) permittivity, (**b**) loss tangent, (**c**) fabricated layered phantom.

The fabricated phantom materials are measured according to the procedure presented in^21^ to determine the permittivity and *tanδ,* and the results are depicted in Fig. 12a,b respectively. To achieve inhomogeneous performance from the phantom, the brain mimicking material is made first and left to set for 5 days in a plastic container. Next, bone mimicking material is poured in the plastic container up to the desired thickness level and left for 5 days to solidify. Finally, the skin layer is formed on top of the bone layer, and again left for 5 days to cure. The resulting inhomogeneous phantom is depicted in Fig. 12c.

**Figure 13:**
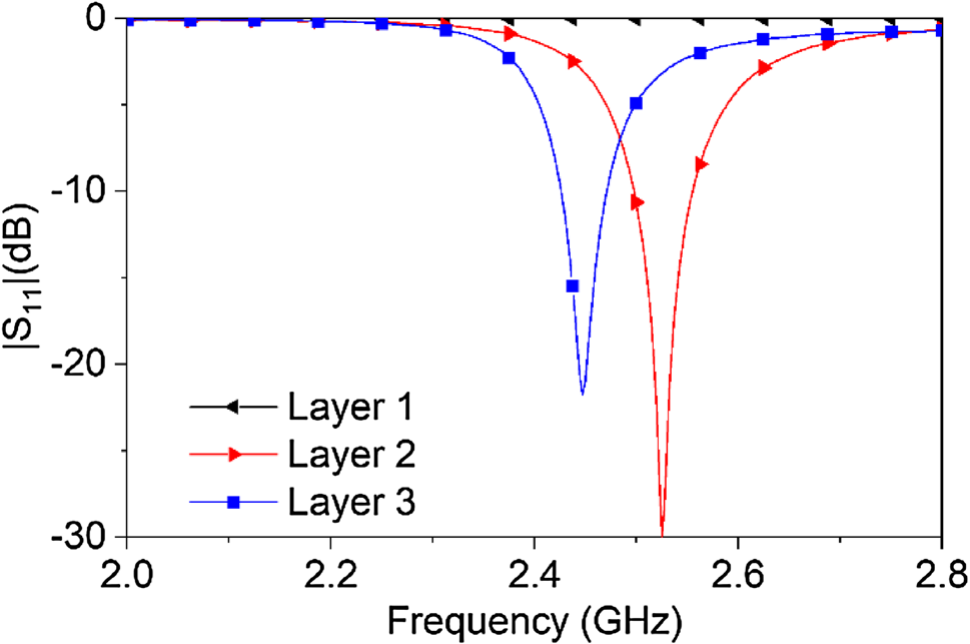
Reflection coefficient of the feeding antenna for addition of different layers on the antenna.

In order to elucidate the development of the antenna, a numerical analysis of its performance is carried out layer by layer while attached to the dielectric loaded waveguide. First, only the ground plane and the probe (*Layer 1*) is examined. The probe feeding technique is utilized by creating a hole through the ground plane, as side feeding technique is not physically suitable for waveguide excitation. The probe is electrically small at 2.45 GHz, and hence no resonance is found at the desired frequency as depicted in Fig. 13. With the addition of the L-probe (*Layer 2*), a resonance is achieved at 2.52 GHz. Once a rectangular patch (*Layer 3*) is implemented 1.9 mm above *Layer 2* to achieve increased directionality at the desired frequency, the resonance of the antenna is shifted to 2.45 GHz with optimal patch dimensions. The 1.9 mm gap between the *Layer 2* and *Layer 3* is realized by using a layer of Rogers RT/duroid 6010 material of 1.9 mm thickness.

**Figure 14:**
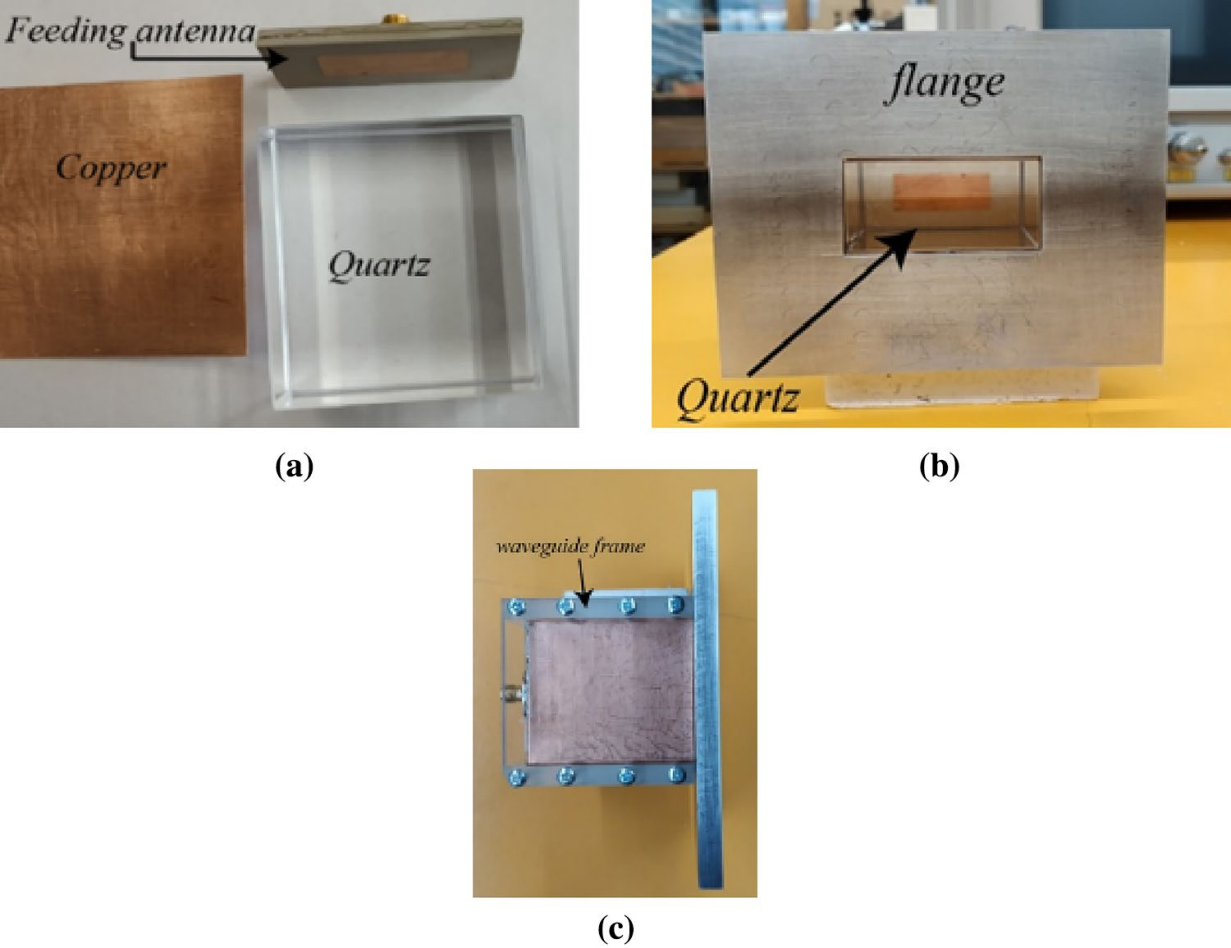
Fabrication of the waveguide prototype: (**a**) before assembly, (**b**) after assembly, (**c**) side view.

A 47 × 23 × 54.65 mm^3^ quartz block with the permittivity of 3.75 and *tanδ* of 0.0004 is selected as the closest suitable material to realize the approximate permittivity of the dielectric loaded waveguide calculated earlier. A copper sheet of 0.5 mm thickness is enfolded around the quartz block to realize the waveguide tube. The constituent components shown in Fig. 14a were assembled, and a waveguide flange with the dimension of 0.25 *λ*_2.45 GHz_ at all sides is realized using aluminium as depicted in Fig. 14b. The side view of the fabricated waveguide is shown in Fig. 14c, showing a waveguide frame made of transparent plastic to hold the constructed waveguide to its rectangular shape.

**Figure 15:**
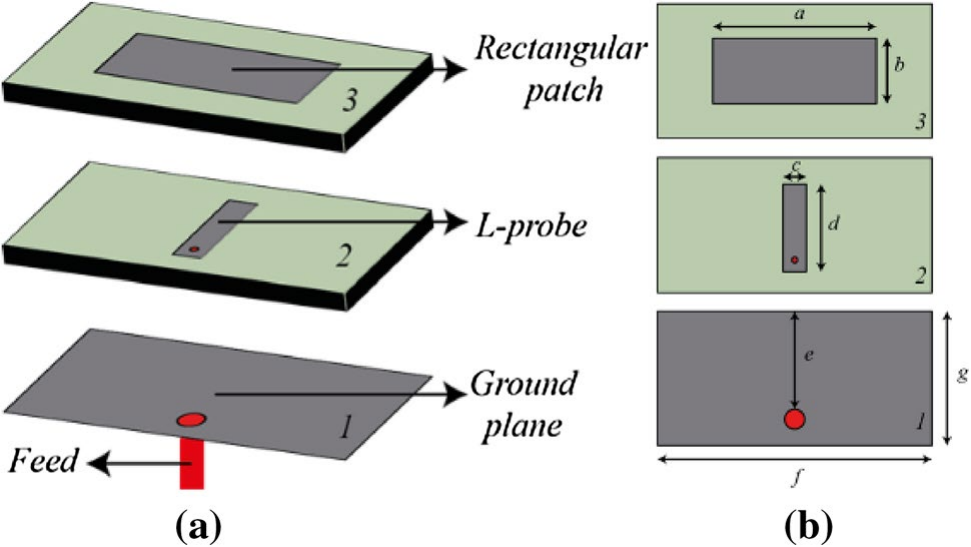
Design of the waveguide feeding antenna (**a**) antenna structure (the gap between the layers is for illustration purpose only, layers 1, 2 and 3 are stacked without any gap in between), (**b**) parameters: *a* = 28, *b* = 11.2, *c* = 4, *d* = 15, *e* = 16.8, *f* = 47, *g* = 23.

**Figure 16:**
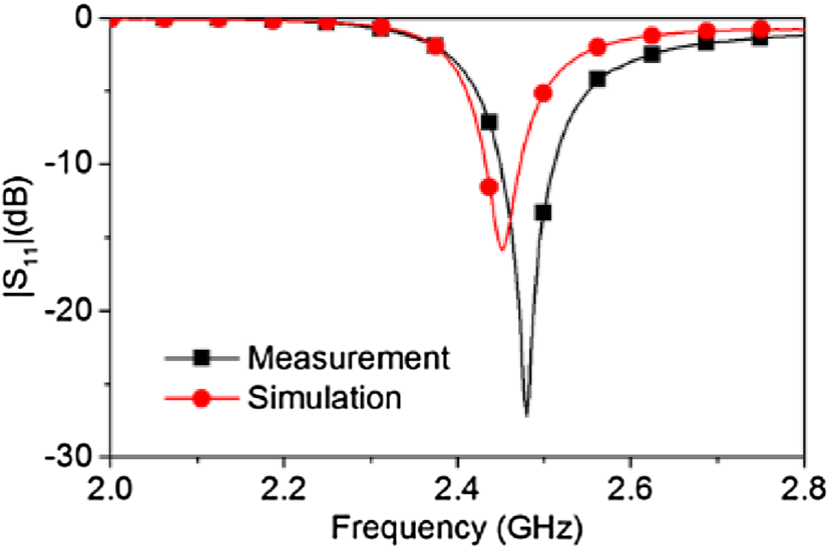
Simulated and measured |S_11_| of the open-ended quartz loaded waveguide.

**Figure 17:**
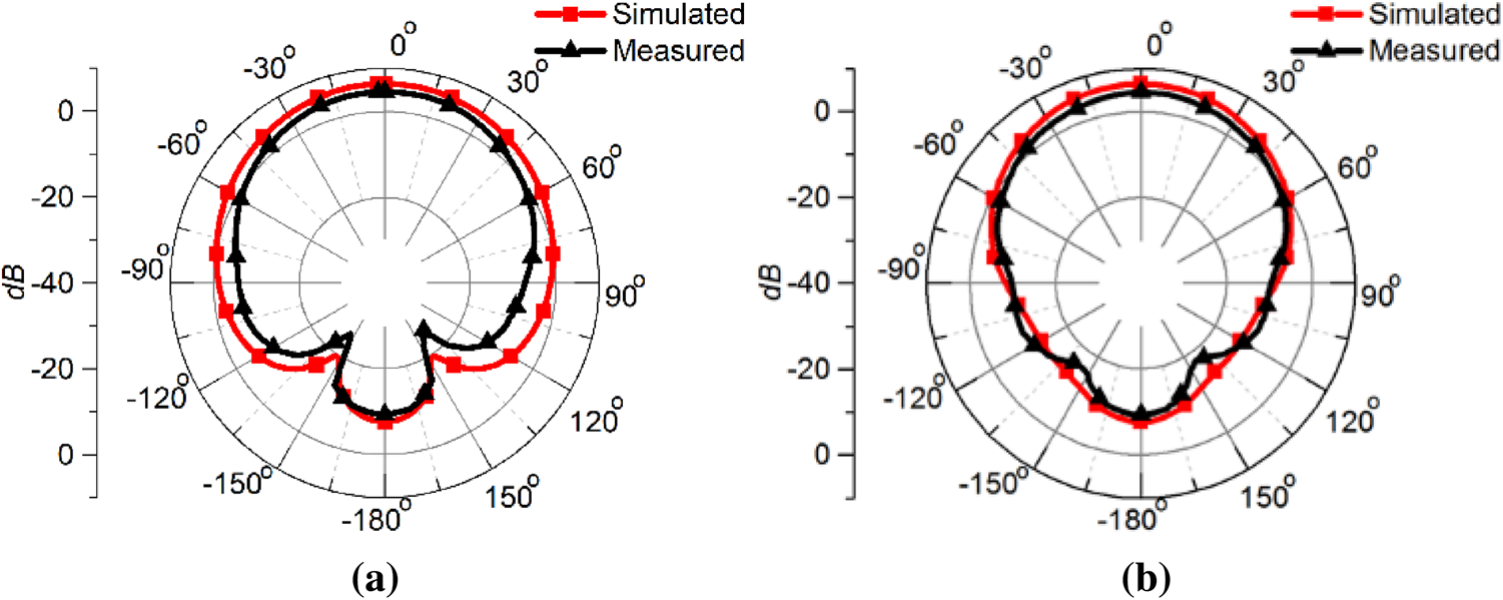
Simulated and measured realized gain patterns of the open-ended quartz loaded waveguide operating at 2.45 GHz in air (**a**) E-plane, (**b**) H-plane.

An antenna feeding technique is utilized to excite the waveguide. Although such a feeding technique lacks broadband performance, the complication of probe feeding a rectangular waveguide via a hole in the fragile quartz block can be avoided by using this method. Moreover, the findings of this research are focused at the specific frequency of 2.45 GHz, so a broadband solution is not required. The physical structure of the antenna is shown in Fig. 15. An L-probe technique is adopted to feed the rectangular patch. Two layers of Rogers RT6010 with relative permittivity of 10.2 and thickness of 1.9 mm is used as the substrate material for the antenna. A 50 Ω SMA connector is used to connect the L-probe and the ground plane. The dimensions of the antenna are optimized to operate at 2.45 GHz while acting as a feed for the dielectric waveguide, in contact with the quartz block and covering one aperture end (as seen in Fig. 14). The simulated and measured |S_11_| response of the dielectric rectangular waveguide with patch antenna feed is depicted in Fig. 16. The |S_11_| performance shows good agreement with only ~ 1% shift in the resonant frequency, which can be attributed to the fabrication tolerances of the waveguide and feed antenna. The simulated and measured realized gain radiation pattern of the open-ended quartz loaded waveguide in free space is depicted in Fig. 17, also exhibiting good agreement in shape with a minor disparity in their maximum values. The simulated radiation patterns show a front to back ratio (FBR) of 14.2 dB, whereas the measured results achieve a FBR of 14 dB. The minor discrepancy in the measured FBR may be partially be attributed to the manual handling of measurement whilst changing the orientation of the waveguide on the turn table of the anechoic chamber.

**Figure 18:**
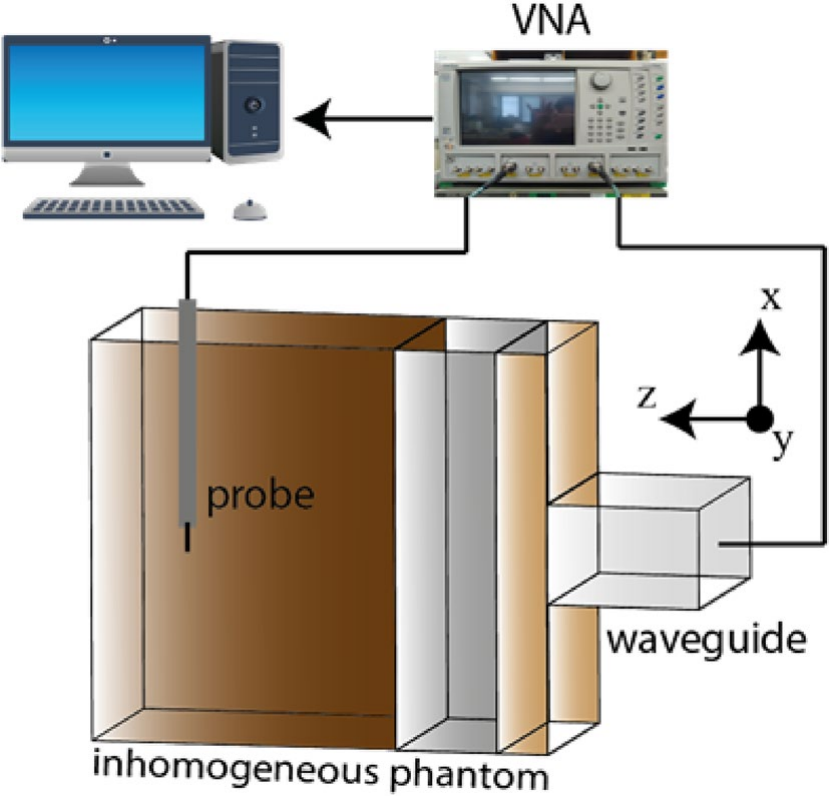
Measurement setup of the power penetration pattern.

A measurement setup is implemented which includes the inhomogeneous head phantom, the rectangular waveguide excitation source, an electrically small monopole probe, an Anritsu (MS4644B) vector network analyser (VNA), and a computer as shown in Fig. 18. The same setup is utilized for both the quartz loaded waveguide and a standard WR 430 waveguide to obtain a comparison. The input power was adjusted to achieve same output power at the aperture for the different waveguides. The power penetration for each waveguide is then measured by placing it against the inhomogeneous human head without any air gap. The electrically small monopole probe is utilized as an E-field probe for radiation pattern measurement in both E- and H-planes. The monopole probe is fabricated from a semi-rigid coaxial cable, and the angle of measurement for the radiation pattern is changed manually by inserting the probe into the human head phantom. The received power is measured via the probe at 50 mm radius from the centre of the waveguide aperture which is mounted flush to the surface of the skin mimicking layer of the inhomogeneous phantom.

**Figure 19:**
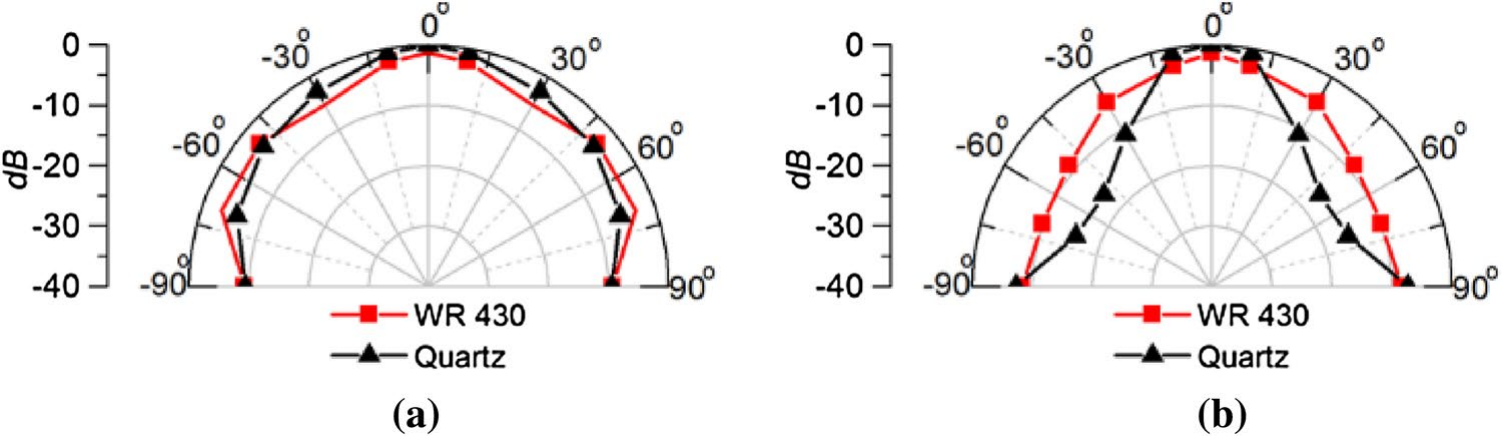
Measured power penetration pattern at a 50 mm radius inside the inhomogeneous phantom for the quartz loaded waveguide and standard WR 430 waveguide at (**a**) E-plane, (**b**) H-plane.

The near field power radiation pattern at both E-plane and H-plane for both the dielectric loaded and standard WR430 waveguide is depicted in Fig. 19. The measured results follow a similar form as predicted in the simulations seen in Fig. 8, with the dielectric loaded waveguide showing enhanced directivity. A power penetration increment of 1.33 dB is achieved utilizing the quartz loaded waveguide at the boresight direction. Moreover, due to the use of the high dielectric material compared to air the waveguide source shrank significantly compared to the standard WR430 waveguide, with an 81.9% decrease in aperture size. The measured power penetration performance validates this wave impedance matching technique for increasing the power penetration into the human head for microwave medical diagnostic systems.

**Figure 20:**
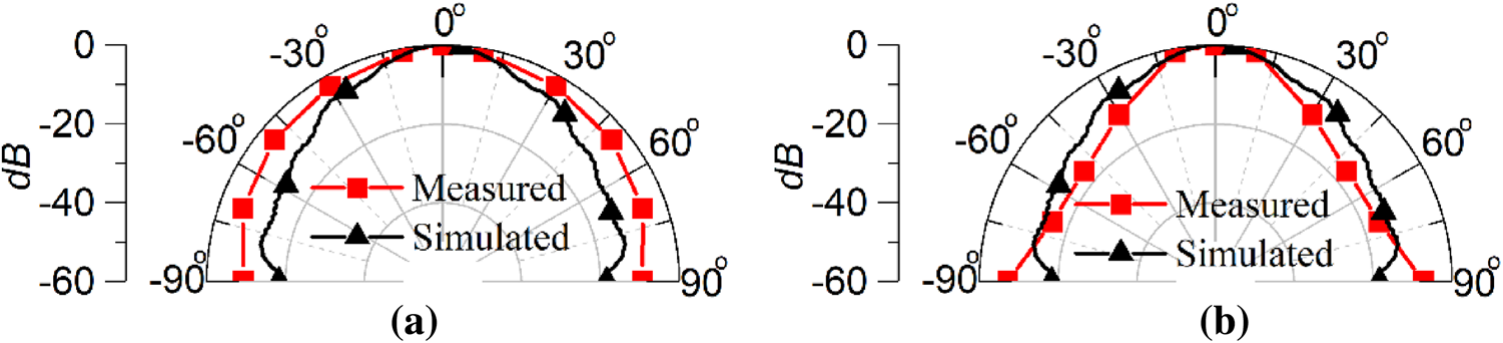
Simulated and measured power penetration patterns inside the human head phantom of the open-ended quartz loaded waveguide operating at 2.45 GHz (**a**) E-plane, (**b**) H-plane.

To aid clarity, a comparison between the simulated and measured power penetration pattern inside the human head phantom is shown in Fig. 20. The measured power penetration pattern in the E-plane is following similar pattern to that predicted in the simulation, although with a slightly broader beam at higher angles from broadside. A half power beamwidth (HPBW) of 30° is exhibited in the simulated E-plane pattern, whereas a 34° HPBW results from the measured pattern. Similarity in the H-plane power penetration pattern is also very good with a HPBW of 30° evident in both the simulated and measured patterns.

## Conclusions

Improvement of the microwave power penetration inside an inhomogeneous human head phantom is achieved by utilizing a dielectric loaded rectangular waveguide for microwave medical diagnostic applications. The combined complex reflection properties of a layered human head model are calculated between skin, bone and brain tissue. A wave impedance matching technique is applied by utilizing a dielectric loaded rectangular waveguide to modify the TE_10_ mode characteristics at 2.45 GHz. An antenna fed Quartz (*SiO*_2_) loaded rectangular waveguide is fabricated along with a layered inhomogeneous human head phantom to measure the power penetration. A measured 1.33 dB power penetration increment is achieved at 50 mm inside the human head phantom by utilizing the designed dielectric loaded waveguide as compared to the standard WR430 waveguide filled with air, and an 81.9% reduction in the aperture size is also attained.
